# Plasma advanced oxidative protein products are associated with anti-oxidative stress pathway genes and malaria in a longitudinal cohort

**DOI:** 10.1186/1475-2875-13-134

**Published:** 2014-04-03

**Authors:** Guicheng Zhang, Oleksii A Skorokhod, Siew-Kim Khoo, Ruth Aguilar, Selma Wiertsema, Augusto J Nhabomba, Tiziana Marrocco, Michelle McNamara-Smith, Maria Nelia Manaca, Arnoldo Barbosa, Llorenç Quintó, Catherine M Hayden, Jack Goldblatt, Caterina Guinovart, Pedro L Alonso, Carlota Dobaño, Evelin Schwarzer, Peter N LeSouëf

**Affiliations:** 1 School of Paediatrics and Child Health, University of Western Australia, c/o 100 Roberts Rd, Subiaco, WA 6008 Perth, Australia; 2 Department of Oncology, University of Torino, Torino, Italy; 3 Centro de Investigação em Saúde de Manhiça (CISM), Maputo, Mozambique; 4 Barcelona Centre for International Health Research (CRESIB, Hospital Clínic, Universitat de Barcelona), Barcelona, Spain; 5 CIBER Epidemiología y Salud Pública (CIBERESP), Barcelona, Spain; 6 School of Public Health, Curtin University, Perth, Australia

**Keywords:** Malaria, Anaemia, Oxidative stress, AOPP, Oxidative pathway genes

## Abstract

**Background:**

Advanced oxidation protein products (AOPP) are newly identified efficient oxidative stress biomarkers. In a longitudinal birth cohort the effects were investigated of genetic polymorphisms in five oxidative pathway genes on AOPP levels.

**Methods:**

This study is part of a three-arm randomized, double-blind, placebo-controlled trial. Three hundred and twelve children were included in the present study with AOPP levels measured at 2.5, 5.5, 10.5, 15 and 24 months of age. Twelve polymorphisms were genotyped in five oxidative stress pathway genes: glutathione reductase (*GSR)*, glutamylcysteine synthetase (*GCLC*), glutathione S-transferase (*GST*) *P1*, haem oxygenase 1 (*HMOX1*) and superoxide dismutase 2 (*SOD2*) in 298 children. There were 284 children assessed for anaemia and clinical malaria infection at the age of 24 months.

**Results:**

Two principal components (PCA1 and PCA2) were derived from the AOPP levels measured at the five time points. PCA1 was significantly associated with anaemia (p = 0.0002), and PCA2 with clinical malaria infection (p = 0.047). In the K-Means Cluster Analysis based on levels of AOPP, children were clustered into two groups: Group A (lower AOPP levels) and Group B (higher AOPP levels). The cluster membership was significantly associated with anaemia (p =0.003) as well as with the *GSR* RS3594 polymorphism (p = 0.037). Mixed linear regression analyses found that the single nucleotide polymorphisms *GCLC* RS10948751 and *HMOX1* RS17885925 were significantly associated with AOPP levels (p = 0.030 and p = 0.027, respectively).

**Conclusion:**

Plasma AOPP levels were predictive for anaemia and oxidative stress markers for clinical malaria infection in two year old children. Several polymorphisms in *GCLC*, *GSR* and *HMOX1* genes were associated with oxidative stress status of these children.

## Background

Oxidative stress is a common pathogenic mechanism underlying the development of many diseases and conditions including malaria infection, in which both the host and the parasite are under its effects. Adapting to the oxidative stress exerted by the host immune response against malaria infection, *Plasmodium falciparum* has developed an elaborate reduction-oxidation (redox) system to maintain adequate antioxidant defence throughout its complex life cycle [1]. In humans, oxidative changes resulting from malaria infection are central to the host protective response against the malaria parasite, and to some of the pathophysiology associated with clinical malaria infection. Enhanced oxidative stress reduces erythrocyte deformability [2,3], contributing to haemolysis, and the development of anaemia [4]. Although oxidative stress, anaemia and malaria infection are closely related in their pathogenic mechanisms, their complex relationships and the functional relevance of oxidative stress genes are poorly understood.

Oxidative pathway genes have been extensively investigated for associations with different phenotypes for their anti-oxidative effects on a range of conditions and diseases [5,6]. Genetic variants in glutathione S-transferase (*GST)* genes have been reported to be associated with susceptibility to many chronic inflammatory conditions and other diseases including malaria [7,8], anaemia [9], asthma [10], allergy [10], chronic obstructive pulmonary disease (COPD) [11], diabetes [12], cardiovascular diseases [12] and cancer [13]. Glutathione reductase (GSR) is also central to cellular antioxidant defence as it reduces oxidized glutathione disulphide (GSSG) to the sulfhydryl form GSH. Glutamate cysteine ligase, also known as gamma-glutamylcysteine synthetase, has a heavy catalytic subunit (GCLC) and is the rate limiting enzyme of glutathione synthesis that plays a crucial role in the intracellular antioxidant defence system. Haem oxygenase 1 (HMOX1), which degrades haem into biliverdin, carbon monoxide and free iron, is an essential enzyme in haem catabolism and protects against oxidative tissue damage. Superoxide dismutase 2 (SOD2) is also an important intracellular antioxidant enzyme. Numerous studies have reported on associations of the genetic variants in *GSR *[11], *GCLC *[14,15], *HMOX1 *[16] and *SOD2 *[17] with oxidative stress related disorders and conditions including malaria [16,18,19].

Evidence of oxidative stress, or an antioxidant status, is usually assessed using biomarkers that reflect the actual oxidative stress in a tissue or the whole body. Plasma proteins, predominantly albumin and fibrinogen, undergo molecular modifications by oxidation which can be measured as advanced oxidation protein products (AOPP), which were first identified by Witko-Sarsat et al. as efficient oxidative stress biomarkers in uraemic patients [20,21]. Further studies confirmed AOPP concentrations as inflammatory markers in several diseases [22,23], but there is no report of any investigation on AOPP in malaria infection prior to this study.

In the AgeMal (Age of exposure and immunity to malaria in infants) collaborative study (ClinicalTrials.gov identifier NCT00231452), the genetic effects were investigated in young children of 12 functionally important polymorphisms in five oxidative pathway genes regarding their functional relevance for oxidative stress status measured as AOPP levels, as well as for the two phenotypes of anaemia and clinical malaria infection. Genetic variants in *GCLC* and *HMOX1* were associated with AOPP concentrations measured at five time points in the first two years of life. The AOPP biomarker was associated with anaemia and clinical malaria infection ascertained at two years of age. There is evidence that oxidative stress pathway genes contribute to the balance between a pro- and anti-oxidant status that is related to the development of anaemia and clinical malaria infection in young children.

## Methods

### Study population

This study is part of the AgeMal project consisting of a three-arm randomized, double-blind, placebo-controlled trial aimed at determining the importance of time of exposure to *P. falciparum* during the first year of life for the development of naturally acquired immunity [24]. The study was conducted in the Manhiça District, Maputo Province, in southern Mozambique, where transmission of *P. falciparum* is perennial with marked seasonality and moderate intensity, and the entomological inoculation rate of 38 infective bites/person/year [25]. The study population has been previously described [24,26,27]. Briefly, HIV-negative pregnant women were recruited during the third trimester of pregnancy and written informed consent was sought to enrol their newborn children in the study. The controlled exposure to *P. falciparum* infection was fulfilled with different protocols for administration of monthly chemoprophylaxis with sulphadoxine-pyrimethamine plus artesunate to the children during the first year of life in a three-arm intervention trial. A total of 312 infants were included in the study, with AOPP measured at 2.5, 5.5, 10.5, 15 and 24 months of age, and > 88% of children analysed at each of the five time points. There were 284 children who had their anaemia and clinical malaria infection status determined at the age of 24 months, and DNA samples were available from 298 children. The study was approved by the National Mozambican Ethics Committee, the Hospital Clínic of Barcelona Ethics Review Committee, the Bioethical Committee of the Torino University School of Medicine, and the Princess Margaret Hospital for Children Ethics Committee (1473/EP) in Perth.

### Genotyping

Genomic DNA was extracted from peripheral blood mononuclear cells by an automated DNA extraction instrument (Autopure LS; QIAGEN, Hilden, Germany). Genetic polymorphisms were selected from the five oxidative stress pathway genes: *GSR*, *GCLC*, *GST P1*, *HMOX1* and *SOD2* based on their potential functional importance and estimated minor allele frequency (>10%) in Africans. Two single nucleotide polymorphisms (SNPs) were genotyped in *GSR*, 3 in *GCLC*, 3 in *GST P1*, 2 SNPs and 1 AG deletion in *HMOX1*, and 1 SNP in *SOD2*. The genomic locations of these polymorphisms in the five genes are shown in Additional file 1. Genotyping of the 11 SNPs and 1 deletion was performed by the Australian Genome Research Facility using the iPLEX assay on the MassARRAY system (Sequenom, San Diego, CA) [28] according to the manufacturer’s instructions.

### Measurement of AOPP concentration in plasma

Spectrophotometric determination of AOPP plasma levels was performed by modification of the Witko-Sarsat’s method in a micro-plate reader [20,29]. Briefly, 200 μl of plasma diluted 1:10 in PBS and chloramine-T standard samples (0-100 μmol/l) were pipetted into a 96-well UV-transparent plate. Eight μl of 1.16 M KI were added to each standard well, followed by 30 μl of acetic acid added two minutes later. The absorbance read at 340 nm against a blank was referred to the standard absorbance. AOPP concentrations were expressed as μmol/l of plasma.

### Definitions of anaemia and clinical malaria infection

A clinical malaria episode was defined as axillary temperature ≥ 37.5°C, or history of fever within the prior 24 h, plus the presence in peripheral blood of *P. falciparum* asexual stage parasites of any density, determined by microscopy following standard quality control procedures [24]. Anaemia was defined as haemoglobin (Hb) < 8 g/dl or packed cell volume (PCV) < 25% (if Hb results were not available), which corresponds to a moderate to severe degree of anaemia.

### Statistical analysis

For the 12 polymorphisms in the five oxidative stress pathway genes, Hardy-Weinberg equilibrium was examined using the online tool [30]. The levels of AOPP at the five time points (2.5, 5.5, 10.5, 15 and 24 months of age) were log transformed to have an approximately normal distribution. Principal component analysis (PCA), which can find a linear combination of variables and in which the combination variables accounts for as much variation in the original variables as possible, was employed to compute the component scores for AOPP levels at the five time points. K-Means Cluster Analysis was used to investigate the cluster membership of individual children based on levels of AOPP at the five time points. Children were clustered into two groups: Group A (lower AOPP levels) and Group B (higher AOPP levels).

The associations between genotypes and PCA scores of AOPP levels were investigated using analysis of variance and independent sample *t* test, when appropriate. The associations of PCA scores of AOPP levels were also investigated with cluster membership, anaemia and clinical malaria infection at the age of 24 months using independent sample *t* tests. Chi-square tests were employed to investigate the associations of genotypes, anaemia, and malaria infection with cluster membership. A mixed linear regression model was further employed to investigate the associations of genotypes with levels of AOPP adjusting for gender and presence/absence of anaemia. Other potential confounding factors were investigated for their association with levels of AOPP. Analyses were conducted using SPSS (PASW Statistics 18). All p-values are two-sided and were considered significant when <0.05.

## Results

### Genotypes

Eleven single nucleotide and 1 insertion/deletion polymorphisms were genotyped in five functionally important oxidative stress pathway genes in 298 young children. Table 1 shows the frequencies of the polymorphisms in these genes. The insertion/deletion variant of RS17883725 in *HMOX1* was not in Hardy-Weinberg Equilibrium so it was excluded from further analyses. There was also complete linkage disequilibrium for the RS10948751 and RS7742367 SNPs in the *GCLC* gene, therefore, only RS10948751 was included in further analyses.

**Table 1:** Frequencies of the 12 polymorphisms in the five oxidative pathway genes

	**Genotypes**	**n**	**%**	**MAF**	**HWE**
**GSR**					
RS1002149(G/T)					
	GG	159	55.0	24.6	0.11
	GT	118	40.8		
	TT	12	4.2		
RS3594 (C/A)					
	CC	243	82.1	9.5	0.73
	CA	50	16.9		
	AA	3	1.0		
**GCLC**					
RS10948751 (A/C)					
	AA	104	35.6	40.6	0.81
	CA	139	47.6		
	CC	49	16.8		
RS1901773 (G/C)					
	GG	69	25.6	48.1	0.46
	CG	142	52.6		
	CC	59	21.9		
RS7742367 (T/C)					
	TT	100	35.7	40.7	0.71
	CT	132	47.1		
	CC	48	17.1		
**GSTP1**					
RS1695 (A/G)					
	AA	81	28.0	47.4	0.81
	AG	142	49.1		
	GG	66	22.8		
RS17593068 (T/G)					
	TT	79	28.0	49.1	0.15
	TG	129	45.7		
	GG	74	26.2		
RS6591256 (A/G)					
	AA	77	26.6	48.3	1.0
	AG	146	50.3		
	GG	67	23.1		
**HMOX1**					
RS11555832 (T/C)					
	TT	107	37.4	0.40	0.31
	CT	129	45.1		
	CC	50	17.5		
RS17883725 (AG/DEL)					
	AG	81	29.2	49.5	0.02
	AG.DEL	118	42.6		
	DEL.DEL	78	28.2		
RS17885925 (T/C)					
	TT	275	92.3	3.9	1.0
	CT	23	7.7		
**SOD2**					
RS4880 (T/C)					
	TT	95	32.3	42.3	0.55
	CT	149	50.7		
	CC	50	17.0		

*MAF* Minor Allele Frequency.

*HWE Hardy-Weinberg Equilibrium*.

### Plasma AOPP

Plasma AOPP levels were measured in 302 children at 2.5 months, 300 at 5.5 months, 296 at 10.5 months, 283 at 15 months and 274 at 24 months of age, showing decreasing levels by age (Table 2). 235 children had plasma AOPP measured at all five time points.

**Table 2:** Levels of AOPP by cross-section

**Months of age**	**n**	**GM (μmol/L)**	**95% CI**	
			**Lower**	**Upper**
2.5	302	352.4	329.5	376.8
5.5	300	263.9	245.4	283.9
10.5	296	207.5	192.3	223.9
15	283	154.0	141.6	167.5
24	274	104.2	96.8	112.1

*GM* Geometric mean.

*CI* Confidence Interval.

### Factor and cluster analysis on plasma AOPP levels

Using PCA two components were derived that explained 50% of the variation of plasma AOPP levels at the five points. The levels of AOPP at the first four time points (2.5, 5.5, 10.5 and 15 months of age) significantly and positively contributed to the first component score (PCA1) that accounted for 30% of the variation of plasma AOPP levels at the five time points. The levels of AOPP at 24 months of age significantly and positively contributed to the second component score (PCA2) that accounted for 20% of the variation of the plasma AOPP levels at the five time points. In addition, the levels of AOPP at 2.5 and 5.5 months of age negatively contributed to the PCA2. Then, the PCA1 was related to plasma AOPP levels before 15 months of age, and the PCA2 was positively correlated with plasma AOPP levels at 24 months of age and negatively correlated with plasma AOPP levels in infants less than 6 months of age. Additional file 1 shows the two component matrix with the plasma AOPP levels at the five time points.

**Figure 1:**
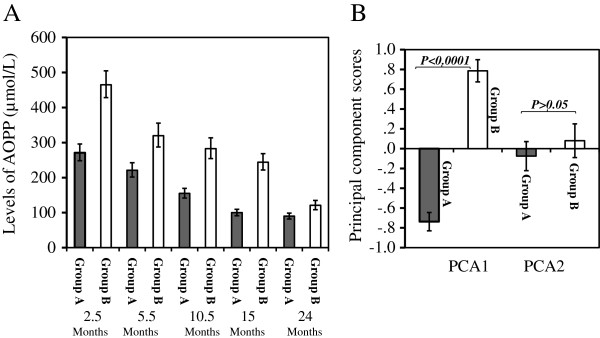
**AOPP levels at the five time points and PCA scores stratified by cluster membership. A**: AOPP levels (geometric mean and 95% confidence intervals) at the five time points; **B**: PCA scores; Cluster membership was clarified using K-Means Cluster Analysis.

Based on the levels of AOPP at the five time points, the children were clustered into two groups: Group A (lower AOPP levels) and Group B (higher AOPP levels). Group A included 161 children and Group B included 151 children. It was expected that the cluster membership would be associated with the levels of AOPP at the five time points as well as to the two PCA components, as the membership was defined from the levels of AOPP. Figure 1 shows the differences in the levels of AOPP (Figure 1A) and in the two principal components (Figure 1B) between children in Group A and Group B. Children in Group A had consistently and significantly lower levels of AOPP at the five time points, and lower scores of PCA1 (p < 0.0001).

### Anaemia and clinical malaria infection at 24 months of age

**Figure 2:**
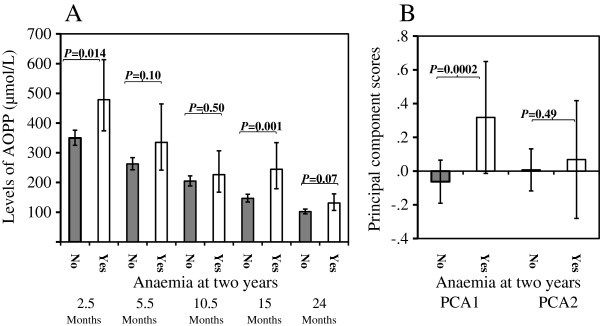
**AOPP levels at the five time points and PCA scores (mean and 95% confidence intervals) stratified by anaemia at two years. A**: AOPP levels (geometric mean and 95% confidence intervals) at the five time points; **B**: PCA scores (geometric mean and 95% confidence intervals); Anaemia was defined at age two years.

**Figure 3:**
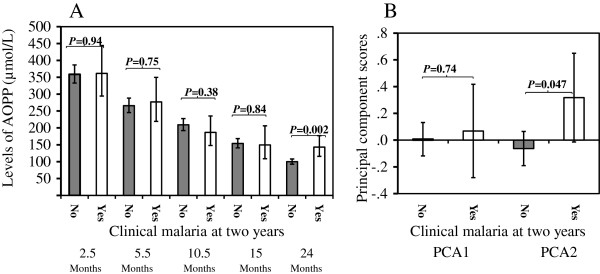
**AOPP levels at the five time points and PCA scores stratified by clinical malaria at two years. A**: AOPP levels (geometric mean and 95% confidence intervals) at the five time points; **B**: PCA scores (mean and 95% confidence intervals); Clinical malaria was defined at age two years.

Among the 284 children who were followed up to the age of 24 months, 23 (8.1%) had anaemia and 32 (11.3%) had clinical malaria infection at this last time point. Generally, children with anaemia at 24 months had higher levels of plasma AOPP at all five time points, with significant differences at ages 2.5 months (p = 0.014) and 15 months (p = 0.001) (Figure 2A). Children with anaemia at 24 months had significantly higher PCA1 scores (p = 0.0002), but not higher PCA2 (Figure 2B). Children with clinical malaria infection at age 24 months did not have significantly higher AOPP levels at ages 2.5, 5.5, 10.5 and 15 months, but had significantly higher AOPP levels at 24 months (Figure 3A). Clinical malaria infection at age 24 months did not associate with PCA1, but was significantly associated with an increased PCA2 (p = 0.047) (Figure 3B).

The associations of the cluster membership were also investigated with anaemia and clinical malaria infection at age 24 months. Children in Group A, that had lower levels of AOPP, had a significantly lower prevalence of anaemia compared with those in Group B (3.4% vs. 13.0%, p = 0.003). The odds ratio of B membership for anaemia was 4.23 (95% confidence interval: 1.53 - 11.7). No association was found between cluster membership and clinical malaria infection at age 24 months.

### Associations of genotypes with the PCA scores, cluster membership and anaemia

**Figure 4:**
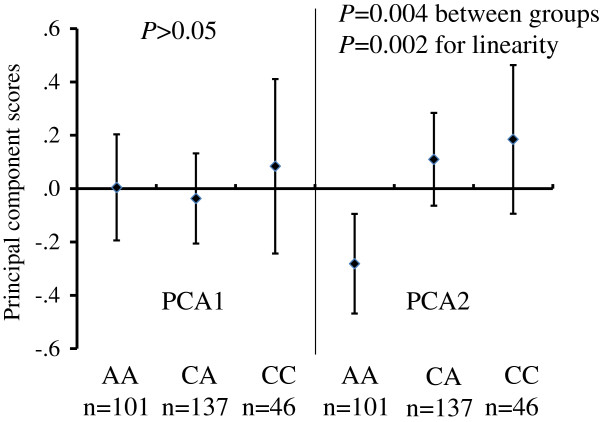
**Associations of the *GCLC *** **RS10948751 genotype with the PCA scores (mean and 95% confidence intervals); Analysis of variance was employed for data analysis.**

**Figure 5:**
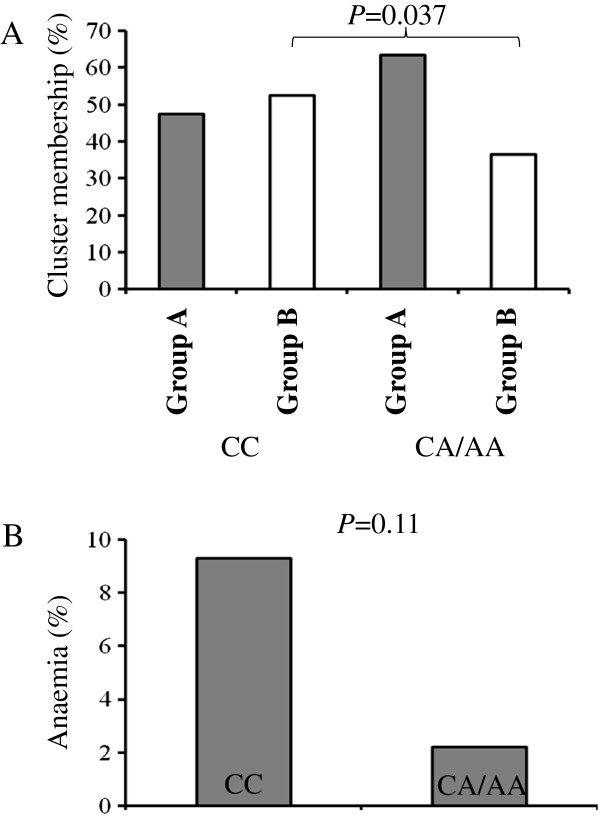
**Associations of the *GSR *RS3594 genotype with the cluster membership and with anaemia; Chi-square tests were used. A**: GSR RS3594 and cluster membership; **B**. GSR RS3594 and anaemia.

The associations of the different genotypes were investigated in the five oxidative pathway genes with the two PCA scores, with the cluster membership and with anaemia. No significant association was found with PCA1, however *GCLC* RS10948751 was significantly associated with PCA2 (Figure 4). No genotypes were associated with the cluster membership except for RS3594 in *GSR* (Figure 5A). The CC homozygotes had a significantly higher percentage of Group B compared with children with CA or AA genotypes (52.6% vs. 36.5%, p = 0.037) (Figure 5A). The CC homozygotes also had a higher percentage of anaemia relative to children with CA or AA genotypes, but this difference was not statistically significant (9.3% vs. 2.2%, p = 0.11) (Figure 5B).

### Mixed linear regression model analyses on the associations of genotypes and AOPP plasma levels

**Figure 6:**
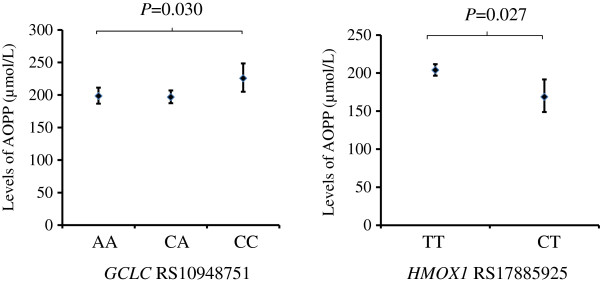
**Associations of plasma AOPP levels (geometric mean and 95% confidence intervals) with the genotypes of *GCLC *** **RS10948751 and *HMOX1 *** **RS17885925; Mixed linear model was employed and adjusted geometric means (adjusted for age and anaemia) were presented.**

Using a mixed linear regression model, the associations of genotypes were investigated with the AOPP plasma levels measured at the five time points. The model showed that the variables age (the five time points) and anaemia (presence/absence) were significantly associated with the plasma AOPP levels, with the presence of anaemia being associated with higher AOPP levels and age associated with decreased AOPP. After adjusting for age and presence/absence of anaemia the SNPs *GCLC* RS10948751 and *HMOX1* RS17885925 were also significantly associated with the levels of AOPP (p = 0.030 and p = 0.027, respectively). Figure 6 shows the levels of AOPP (adjusted for age and anaemia) for the different genotypes of these two SNPs. In the regression model analysis, the effects of gender, maternal malaria infection and intervention were not significantly associated to the AOPP levels.

## Discussion

This is the first study to investigate the complex relationships between oxidative stress pathway genes, the oxidative stress biomarker AOPP, anaemia and clinical malaria infection in a longitudinal cohort of young children. The findings in this study are: (1) Plasma AOPP is a valuable oxidative stress biomarker of anaemia and clinical malaria infection in children two years old living in a malaria endemic area; (2) Preceding levels of AOPP predict to some extent the development of anaemia in children two years old; and (3) Variants in *GCLC*, *GSR* and *HMOX1* genes are associated with the host oxidative stress status, which was evidenced by their associations with AOPP levels. This study provides valuable data to explain the complex oxidative stress phenomena in children regarding anaemia and clinical malaria infection.

AOPP are biomarkers for oxidative stress, and were first detected in the plasma of chronic uraemic patients [20]. In addition, AOPP have been investigated for associations with many other chronic conditions such as allergic rhinitis [31], severe obesity [32,33], inflammatory bowel disease [34], atherosclerosis [35], coronary artery disease [36], ulcerative colitis [37], and many others [38-42]. There is evidence that AOPP are both biomarkers of an imbalance between pro-oxidants and anti-oxidants, and mediators of inflammation [22,41,43]. In this study conducted on a longitudinal cohort of children enrolled at birth, it has clearly been demonstrated that children with anaemia at age two years had increased plasma AOPP levels at ages 2.5, 5.5, 10.5, 15 and 24 months. Moreover, based on the levels of AOPP, children were stratified into two groups using K-Means Cluster techniques, and children in Group B (higher AOPP levels) had more than four times increased risk for anaemia at age two years relative to children in Group A (lower AOPP levels). The association observed between AOPP levels measured just after birth and the presence of anaemia at age two years suggests that the early oxidative stress burden predicts the later development of anaemia in young children living in a malaria endemic area. Children with clinical malaria infection at age two years had concurrently higher levels of AOPP, which may have resulted from the simultaneous interdependence of oxidative stress with malaria infection. In addition, clinical malaria infection at age two years was associated with a higher score for PCA2 that was positively correlated with AOPP levels at age 24 months, and negatively correlated with AOPP levels at ages 2.5 and 5.5 months. Since PCA2 only explained 20% of the variation of AOPP levels at the five time points, there was insufficient evidence that the early levels of AOPP at 2.5 and 5.5 months, possibly related to early malaria exposure, were associated with a protective effect against later clinical malaria. Moreover, the raw analysis on the association between AOPP levels at 2.5 and 5.5 months did not support this relationship. Interestingly however, the RBC oxidative stress marker 4-hydroxynonenal-conjugates, was recently reported to be associated with malaria susceptibility in young children [27]. Therefore, more studies, using AOPP as biomarkers are indicated to further elucidate the relationship between early oxidative burden, and later susceptibility to clinical malaria. However, the data in the present study showed that AOPP levels were an early predictor for anaemia and a simultaneous oxidative stress biomarker of clinical malaria in two years old children.

The main goal of this study was to investigate the associations of 12 functionally important polymorphisms in five oxidative stress pathway genes with plasma AOPP levels. One polymorphism which violated the Hardy-Weinberg equilibrium and one redundant polymorphism were excluded, thus only 10 were included in the analyses. To circumvent multiple tests, the relationships were examined of these 10 polymorphisms with the two PCA scores derived from the AOPP levels at the five time points. The *GCLC* SNP RS10948751 was associated with PCA2, and the further mixed linear regression model analysis confirmed the association of this SNP with plasma AOPP levels. There are no reports investigating associations of variations of the *GCLC* gene with susceptibility to malaria or with the biomarker AOPP. However, a few studies have reported that the SNP -129 C/T and the TNR (GAG trinucleotide repeat) polymorphism in the *GCLC* gene are associated with several clinical phenotypes such as type 1 diabetes [44], chronic beryllium disease [45], depression [46] and lung function and growth [47]. This study provides new evidence that sequence variations of the glutamate cysteine ligase gene are related to the anti-oxidative stress-response. Consistent with a previous study [45] the polymorphism-associated variation in the AOPP levels may increase pro-inflammatory phagocyte activity thus inducing lipid-peroxidation activity with subsequent oxidative plasma and RBC membrane modifications [48], decreased RBC filterability [3], enhanced RBC phagocytosis [49] and defective erythropoiesis [48]. These cellular processes may partly explain the associations found between polymorphisms in this gene and anaemia. In addition, one variant in the *GSR* gene (RS3594) was associated with cluster membership based on AOPP levels measured longitudinally during the first two years of life. Polymorphisms in the GSR gene have been associated with postmenopausal bone mineral density values [17] and COPD [11]. This study provides evidence for the functional importance of polymorphisms in the GSR gene in relation to oxidative stress in an inflammatory disease. In case of an association between the GSR polymorphisms and a low anti-oxidant glutathione reducing activity, increased AOPP values would be the direct consequence of any inflammatory response or oxidation by the malaria parasite growing in RBCs. Indeed, *P-falciparum*-parasitized RBCs with these *GSR* phenotypes were reported to show increased *in-vitro* phagocytosis rates due to membrane oxidations [50]. Similar mechanisms might play a role *in vivo* and explain the association of the *GSR* polymorphism with anaemia. A few studies have investigated the associations of the *HMOX1* gene polymorphisms with malaria susceptibility in humans [16,19,51]. A microsatellite polymorphism (GT)n was found to be associated with HMOX1 expression and development of symptomatic or severe malaria [16,19]. The present study found that the *HMOX1* RS17885925 polymorphism was associated with AOPP levels. However, considering the linkage disequilibrium between polymorphisms in this gene, the effect of *HMOX1* RS17885925 on the AOPP levels may be attributed to other intragenic variants [16,19].

The main limitation of this study was that the longitudinal cohort had a small sample size (less than 300 children at age two years). As the subjects were recruited from a general African community, the prevalence of anaemia and clinical malaria infection was less than 12%, with only 23 anaemic children and 32 children with clinical malaria infection at age two years. This study, therefore, did not have sufficient statistical power to investigate the associations of the different genotypes with the anaemia and clinical malaria infection phenotypes. However, AOPP plasma levels were longitudinally measured, and this has provided a comprehensive assessment of the children’s oxidative status and allowed a systematic examination of the complex interrelationships between AOPP plasma levels, polymorphisms in several oxidative stress pathway genes and anaemia and clinical malaria infection in the longitudinal cohort.

In conclusion, the present study has identified several polymorphisms in *GCLC*, *GSR* and *HMOX1* genes that are associated with oxidative stress status in the blood plasma of two-year old children from a malaria endemic region. In addition, plasma AOPP is an oxidative stress marker for clinical malaria in these children and that AOPP levels are predictive for anaemia.

## Competing interest

The authors do not have any commercial or other association that might pose a conflict of interest. The funding sources did not have any involvement in study design, collection, analysis and interpretation of data, writing of the report, or in the decision to submit the paper for publication. The researchers are independent from the funders.

## Authors’ contributions

GZ, ES, PNL, CD, PLA, JG contributed to conception and design of the study; TM, RA, OAS, ES, KSK, SW, CMH, AN, MNM, AB, CG contributed to acquisition of data; SW, KSK, CMH contributed to selection of genes and polymorphisms; GZ, MSM, LQ analyzed the data; all authors contributed to interpretation of data; GZ drafted the article; all authors revised the article critically for important intellectual content; all authors read and gave final approval of the version to be published.

## Supplementary Material

Additional file 1: Table S1 Polymorphisms in the five oxidative pathway genes. **Table S2.** Component matrix of levels of AOPP at five times with two principal component scores. Click here for file
