# Supplementary material for: Plasma advanced oxidative protein products are associated with anti-oxidative stress pathway genes and malaria in a longitudinal cohort
Source: Malar J. 2014 Apr 3;13:134. doi: 10.1186/1475-2875-13-134 (PMC4230024; doi:10.1186/1475-2875-13-134)
Supplement: Additional file 1: Table S1 — Polymorphisms in the five oxidative pathway genes. Table S2. Component matrix of levels of AOPP at five times with two principal component scores. [file 1475-2875-13-134-S1.docx]

**PLASMA ADVANCED OXIDATIVE PROTEIN PRODUCTS ARE ASSOCIATED WITH OXIDATIVE STRESS PATHWAY GENES AND MALARIA IN A LONGITUDINAL COHORT**

Zhang, Guicheng^1,5^; Skorokhod, Oleksii A^2^; Khoo, Siew-Kim^1^; Aguilar, Ruth^3,4^; Wiertsema, Selma^1^; Nhabomba, Augusto^4^; Marrocco, Tiziana^2^; McNamara-Smith, Michelle^1^; Manaca, M. Nelia^4^; Barbosa, Arnoldo^4^; Quintó, Llorenç^3^; Hayden, Catherine M^1^; Goldblatt, Jack^1^; Guinovart, Caterina^3,4^; Alonso, Pedro, L ^3,4^; Dobaño, Carlota^3,4^, Schwarzer, Evelin^2^*, LeSouëf, Peter N^1^*

* Both authors contributed equally

1. School of Paediatrics and Child Health, University of Western Australia, Perth, Australia
2. University of Torino, Department of Oncology, Torino, Italy
3. Barcelona Centre for International Health Research (CRESIB, Hospital Clínic, Universitat de Barcelona), Barcelona, Spain
4. Manhiça Health Research Center (CISM), Manhiça, Mozambique
5. School of Public Health, Curtin University, Perth, Australia

Additional file 1: Table S1 Polymorphisms in the five oxidative pathway genes

| Gene | SNP | Region | Amino Acid Change | Chr. Position |
| --- | --- | --- | --- | --- |
| **Glutathione Reductase (*GSR*) 8p21.1** | | | | |
| RS1002149 | G/T | 5' near |  | 30585738 |
| RS3594 | C/A | 3' UTR |  | 30535660 |
| **Glutamate-cysteine ligase catalytic subunit (GCLC) 6p12** | | | | |
| RS10948751 | A/C | 3' near |  | 53469359 |
| RS1901773 | G/C | 3' near |  | 53469948 |
| RS7742367 | T/C | 3' near |  | 53469235 |
| **Glutathione S-transferase Pi-1 (*GSTP1*)** [11q13](http://www.ncbi.nlm.nih.gov/entrez/query.fcgi?db=gene&cmd=Retrieve&dopt=full_report&list_uids=2950) | | | | |
| RS1695 | A/G | Exon 5 | IIe 105Val | 67352689 |
| RS17593068 | T/G | 5' near |  | 67360932 |
| RS6591256 | A/G | 5' near |  | 67349899 |
| **Heme oxygenase 1 (Hmox1)** [22q12-22q13.1](http://www.ncbi.nlm.nih.gov/entrez/query.fcgi?db=gene&cmd=Retrieve&dopt=full_report&list_uids=3162) | | | | |
| RS11555832 | T/C | 3’ UTR |  | 35789869 |
| RS17883752 | AG/Del | 5' near |  | 35776258 |
| RS17885925 | T/C | intron |  | 35779223 |
| **Manganese superdioxide dismutase 2 (SOD2)** [6q25.3](http://www.ncbi.nlm.nih.gov/Omim/getmap.cgi?l147460) | | | | |
| RS4880 | T/C | Exon 2 | Val16Ala | 160113872 |

| Additional file 1: Table S2 Component matrix of levels of AOPP at five times with two principal component scores | | |
| --- | --- | --- |
|  | Component | |
|  | PCA1 | PCA2 |
| AOPP at 2.5 months | .607 | -.318 |
| AOPP at 5 months | .500 | -.534 |
| AOPP at 10.5 months | .630 | .169 |
| AOPP at 15 months | .592 | .219 |
| AOPP at 24 months | .295 | .760 |
| Extraction Method: Principal Component Analysis (PCA) with two components extracted. | | |
|  | | |
